# Intramedullary nailing versus cemented plate for treating metastatic pathological fracture of the proximal humerus: a comparison study and literature review

**DOI:** 10.1186/s10195-023-00721-7

**Published:** 2023-08-24

**Authors:** Karl Wu, Ting Lin, Cheng-Han Lee

**Affiliations:** 1 Department of Physical Education and Sport Sciences, National Taiwan Normal University, Taipei, Taiwan; 2 Department of Materials and Textiles, Oriental Institute of Technology, New Taipei City, 220 Taiwan; 3 Department of Orthopedic Surgery, Far Eastern Memorial Hospital, No. 21, Sec. 2, Nanya S. Rd., New Taipei City, 220 Taiwan (R.O.C.); 4 Department of Nursing, Far Eastern Memorial Hospital, New Taipei City, Taiwan; 5 Department of Orthopedic Surgery, Nantou Hospital, Ministry of Health and Welfare, Nantou, Taiwan

**Keywords:** Intramedullary nailing, Proximal humerus, Pathological fracture, Metastatic bone tumor

## Abstract

**Background:**

Pathological fracture of the humerus causes severe pain, limited use of the hand, and decreased quality of life. This study aimed to compare the outcomes of intramedullary nailing and locking plate in treating metastatic pathological fractures of the proximal humerus.

**Methods:**

This retrospective comparison study included 45 patients (22 male, 23 female) with proximal humerus metastatic pathological fractures who underwent surgical treatment between 2011 and 2022. All data were collected from medical records and were analyzed retrospectively. Seventeen cases underwent intramedullary nailing plus cement augmentation, and 28 cases underwent locking plate plus cement augmentation. The main outcomes were pain relief, function scores, and complications.

**Results:**

Among 45 patients with mean age 61.7 ± 9.7 years, 23 (51.1%) had multiple bone metastases, and 28 (62.2%) were diagnosed with impending fractures. The nailing group had significantly lower blood loss [100 (60–200) versus 500 (350–600) ml, *p* < 0.001] and shorter hospital stay (8.4 ± 2.6 versus 12.3 ± 4.3 days, *p* < 0.001) than the plating group. Average follow-up time of the nailing group was 12 months and 16.5 months for the plating group. The nailing group had higher visual analog scale (VAS) scores than the plating group, indicating greater pain relief with nailing [7 (6–8) versus 6 (5–7), *p* = 0.01]. Musculoskeletal Tumor Society functional scores [28 (27–29) versus 27 (26.5–28.5), *p* = 0.23] were comparable between groups. No complications, local recurrence, or revision surgery were reported until the last follow-up in either group. However, one case in the plating group had a humeral head collapse and fragmentation without needing revision surgery.

**Conclusions:**

Intramedullary nailing with cement augmentation is a viable option for treating proximal humerus metastatic pathological fracture, providing rigid fixation and better pain relief resulting in earlier mobility to optimize functional outcomes. Less invasive procedure with less blood loss and shorter hospital stay also benefits patients.

*Level of evidence* Level II.

*Trial registration statement* Not applicable.

## Introduction

Metastatic bone disease is the leading cause of destructive bone lesions in adults. The humerus is the second most common long-bone site for metastatic bone disease after the femur [1]. Proximal third and diaphysis are frequent sites for bone metastases in the humerus. Lesions in the distal third of the humerus are less common and usually found to be lung, myeloma, or renal carcinoma [2]. Although the humerus is not a weight-bearing bone, pathological fracture of the humerus still causes severe pain, limited use of the hand, and decreased quality of life. Surgical stabilization of symptomatic impending or pathologic fractures is often indicated in patients with average general conditions and long life expectancy. Treatment options depend on individual circumstances, such as the location and extent of the lesion, bone quality, general condition, and the patient’s life expectancy. The goal of treatment is to restore an anatomic limb length and alignment and create a stable construct for early mobility and daily use [3].

Various surgical methods have been established for the management of pathological fracture of the humerus, including intramedullary nailing, plating, and endoprosthesis. For lesions in the proximal humerus, the mainstay treatment of choice is endoprosthetic replacement for the intraarticular uncontained lesion, lesion at the humeral head, or lesion at the proximal metaphysis [3, 4]. Endoprosthetic reconstruction has the advantages of resistance-rotation and bending, good analgesic effect, and local tumor control and stability. However, dysfunction is a problem owing to the rotator cuff sacrificing stability for mobility, inserting the greater tuberosity of the humerus [4]. Plate fixation with cement augmentation has been successfully used in treating proximal metaphysis-contained lesions [5]. Choi et al. reported using intramedullary nailing in the proximal humerus pathological fracture [6].

Intramedullary nailing was at one time considered inappropriate for treating proximal humerus pathological fractures because it could not provide rigid fixation after tumor curettage. The thin cortex and mainly cancellous bone in the proximal metaphysis of the humerus did not allow rigid fixation from osteosynthesis. However, with modern design, interlocking humeral nails have been successfully used in treating proximal humerus fracture [6, 7]. This retrospective study aimed to evaluate and compare outcomes of intramedullary nailing and locking plates in treating metastatic proximal humerus pathological fractures.

## Patients and methods

### Patients

**Fig. 1:**
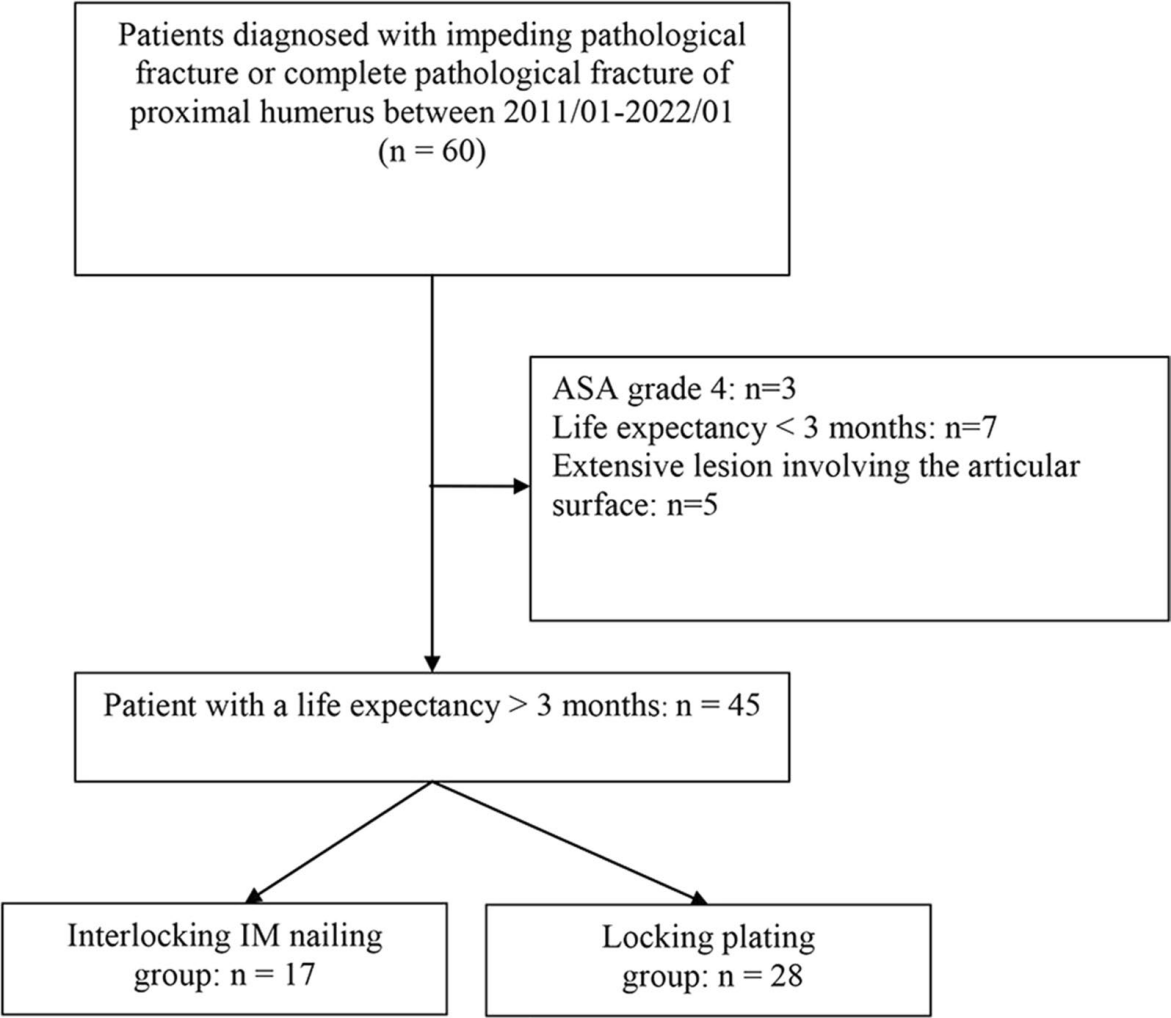
Flowchart of patient selection

This retrospective comparative study included consecutive patients with complete pathological fractures or metastatic impending pathological fracture of the proximal humerus who were treated surgically in our institution between January 2011 and January 2022. Pathological fractures of the proximal humerus were defined as metastatic lesions in the humeral neck or proximal humerus leading to imminent or complete fractures. Prior to undergoing surgery, all patients consulted with an oncologist and anesthesiologist regarding perioperative risks and life expectancy. Inclusion criteria were: (1) symptomatic impending or complete pathological fracture and (2) life expectancy of more than 3 months. Exclusion criteria were: (1) life expectancy of less than 3 months, (2) preoperative American Society of Anesthesiology (ASA) grade 4, (3) extensive lesion involving the articular surface. When selecting the implant, the histology of the primary tumor was not considered. A total of 45 patients were included for final analysis, including 13 and 12 patients with multiple and solitary bone metastases, respectively. Seventeen cases were treated with intramedullary nailing plus cement augmentation, while 28 cases were treated with locking plate plus cement augmentation, depending on physician judgment (Fig. 1). All cases were treated based on the same criteria. The differences lie in the early stages of treating pathological humerus fractures, before the widespread use of the nailing system, when plates were commonly employed as the “standard procedure.” However, as our experience using humeral nails for the treatment of fractures progressed and we became increasingly proficient, this technique was gradually applied more often to these pathological fracture cases. A minimum follow-up period of 12 months was achieved for those who survived.

### Ethical considerations

The study protocol was reviewed and approved by the Institutional Review Board. Because of the retrospective design of the study and anonymity of the patients, signed informed consent was waived.

### Surgical procedure: intramedullary nailing with cement augmentation

The patient was semi-sitting, and the surgery was performed under general anesthesia. A limited anterolateral approach was made for tumor curettage and nail insertion. The axillary nerve was identified and protected whenever possible. All gross tumors were removed as much as possible. Curettage through the fracture gap usually resulted in a cortical bone defect, enough for cement impaction when the fracture was complete. A cortical window was made in the thinnest cortex wall on the anterolateral proximal humerus when the fracture was impending. After meticulous curettage of gross tumors, local adjuvant therapy of 95% alcohol was applied. Then the nail (DePuy Synthes, MultiLoc Humeral Nails, Raynham, MA, USA) was inserted in an antegrade manner after series remaining to desired width. The nail should be long enough to cover the entire humerus. The nail-end position was carefully checked and buried about 5 mm below the articular surface of the humerus head because the need for nail removal in the future was not expected and the goal was to avoid nail impingement to rotator cuff tendons. For fixation, we usually applied two or three proximal screws and one or two distal screws. The bone defect was filled by poly(methyl methacrylate) bone cement after nail fixation. Early range of motion was encouraged the next day after surgery. A rehabilitation program such as passive stretching or gravity-resistant exercise was arranged about 1 week later. Adjuvant external beam radiation with 3000–3500 Gy was performed for most patients. Since reaming was used for better size nail insertion, radiotherapy was deemed necessary for local tumor control owing to the risk of tumor dissemination after reaming.

### Surgical procedure: humerus plating with cement augmentation

The surgery was performed applying the same basic principles as nailing. An anterolateral approach was made to directly expose the fracture site or gross tumor part with the patient in a semi-sitting position. The axillary nerve wound was also protected whenever possible. After meticulous tumor curettage, local adjuvant therapy of 95% alcohol was applied. Then poly (methyl methacrylate) (PMMA) cement was infilled into the skeletal defect. A proximal humerus locking plate (DePuy Synthes, Inc., Philos^®^ System, Raynham, MA, USA) was placed and inserted with appropriate size screws for fixation. The plating group shared the same postoperative protocol as the nailing group. Adjuvant external beam radiation with 3000–3500 Gy was performed for most patients.

### Outcomes

The primary outcomes were pain relief and functional status, and the secondary outcome was perioperative complications. Visual analog scale (VAS) scores were evaluated to monitor pain improvement from before surgery to 1 week and 1 month after surgery. Functional status was assessed by the shoulder joint range of motion such as forward flexion and abduction 1 month after surgery, and the Musculoskeletal Tumor Society rating scale (MSTS) and Karnofsky performance status scale were used 3 months postoperatively. Perioperative parameters such as operative time, blood loss, and hospital length of stay were also recorded. Possible complications included radial nerve injury, shoulder impingement, infection, implant failure, or revision surgeries.

### Statistical analysis

Normally distributed continuous data are presented as mean ± standard deviation (SD) and evaluated by Student’s *t*-test; non-normally distributed continuous data are presented as median and interquartile range (IQR) and analyzed by Wilcoxon rank-sum test. Shapiro–Wilk test for normality was used for testing normal distribution of variables. Categorical data are presented as counts and percentages and analyzed by chi-square or Fisher’s exact test. Kaplan–Meier survival analysis was used to evaluate the reliability of each group and the overall survival of patients. All *p*-values are two-sided, and *p* < 0.05 was considered statistically significant. All statistical analysis was performed using SAS software version 9.4 (SAS Institute Inc., Cary, NC, USA).

### Literature review

The review was performed in accordance to PRISMA guidelines. Medline, and Cochrane, and Google Scholar database were searched until 15 November 2022 for studies that evaluated effects of surgical treatments in treating metastatic pathological fracture of the proximal humerus. The following search terms were used: proximal humerus, metastatic pathological fracture, surgery, endoprosthesis replacement, locking plate, and nailing. Included studies were those that evaluated outcomes of endoprosthesis replacement, plating with cement, or intramedullary nailing with cement in patients diagnosed with metastatic pathological fractures of proximal humerus. Letters, editorials, comments, and case reports were excluded from the systemic review. Studies were identified and manually searched by at least two independent reviewers. When there was uncertainty regarding eligibility, a third reviewer was consulted. The following information and data were extracted from included studies: name of first author, year of publication, surgical mode, number of participants, participants’ age, postoperative function level, pain control level, complications, tumor recurrence, reoperation.

## Results

The baseline demographic and clinical characteristics of the study population stratified by surgery type are presented in Table 1. Patients’ mean age was 61.7 ± 9.7 years, 22 (48.9%) were male, 23 (51.1%) had multiple bone metastases, and 28 (62.2%) were diagnosed with impending fractures. Patients who received nailing were older (66.6 ± 9.8 versus 58.7 ± 8.5 years, *p* = 0.006) than those who received plating. No significant differences in other characteristics were observed between groups.

**Table 1:** Patients’ demographic and clinical characteristics stratified by surgical procedure

Variable	Total	Nailing (*n* = 17)	Plating (*n* = 28)	*p*-Value
Age, years	61.7 ± 9.7	66.6 ± 9.8	58.7 ± 8.5	**0.006**
Sex				0.848
Male	22 (48.9)	8 (47.1)	14 (50.0)	
Female	23 (51.1)	9 (52.9)	14 (50.0)	
Site				0.977
Left	16 (35.6)	6 (35.3)	10 (35.7)	
Right	29 (64.4)	11 (64.7)	18 (64.3)	
Lesion site in the humerus				0.908^a^
Head and neck	17 (37.8)	6 (35.3)	11 (39.3)	
Neck	24 (53.3)	9 (52.9)	15 (53.6)	
Head, neck to shaft	4 (8.9)	2 (11.8)	2 (7.1)	
Bone metastases site				0.848
Solitary	23 (51.1)	9 (52.9)	14 (50.0)	
Multiple	22 (48.9)	8 (47.1)	14 (50.0)	
Diagnosis				0.789
Pathological fracture	17 (37.8)	6 (35.3)	11 (39.3)	
Impending fracture	28 (62.2)	11 (64.7)	17 (60.7)	
Primary tumor				0.368^a^
Lung	15 (33.3)	6 (35.3)	9 (32.1)	
Breast	10 (22.2)	3 (17.6)	7 (25.0)	
HCC	9 (20.0)	4 (23.5)	5 (17.9)	
Lymphoma	1 (2.2)	0 (0.0)	1 (3.6)	
Melanoma	1 (2.2)	1 (5.9)	0 (0.0)	
Cholangiocarcinoma	1 (2.2)	1 (5.9)	0 (0.0)	
Bladder	1 (2.2)	1 (5.9)	0 (0.0)	
Prostate	7 (15.6)	1 (5.9)	6 (21.4)	

*P*-Value < 0.05 is shown in bold

HCC, hepatocellular carcinoma

a. Fisher’s exact test

**Fig. 2:**
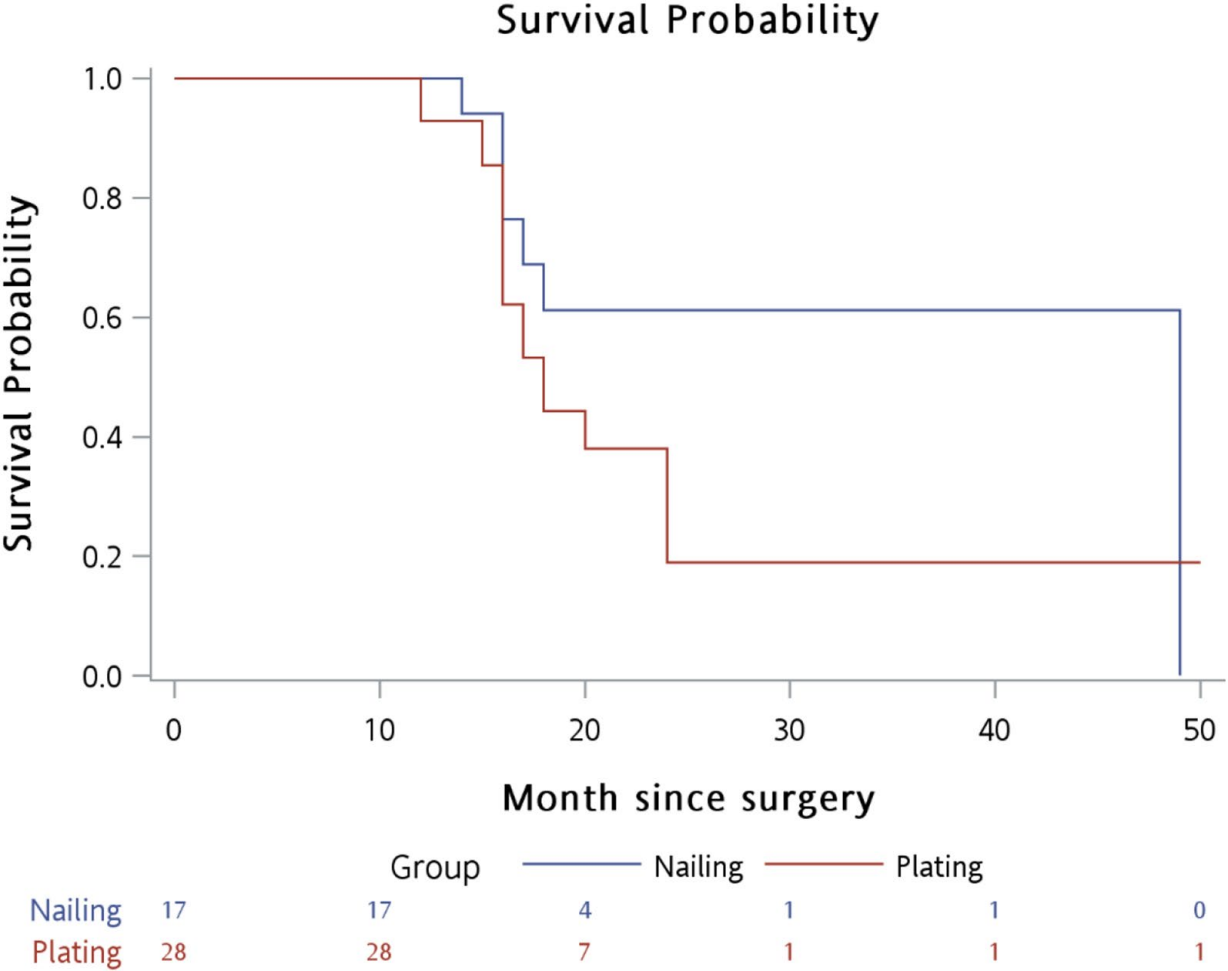
Kaplan–Meier survival analysis for overall survival between surgical procedures (log-rank *p*-value = 0.215)

The outcomes of the surgical procedures are compared in Table 2. In the nailing group, blood loss (median (IQR): 100.0 (60.0–200.0) versus 500.0 (350.0–600.0) ml, *p* < 0.001) and length of hospital stay (8.4 ± 2.6 versus 12.3 ± 4.3 days, *p* < 0.001) were significantly lower than those in the plating group. No perioperative morbidity such as embolism or cardiovascular events occurred in either group. A significant difference was found in VAS scores 1 month postoperatively between the nailing and plating groups [median (IQR): 2.0 (0.0–2.0) versus 2.0 (2.0–2.5), *p* < 0.002]. The reduction in VAS scores in the nailing group was greater than that in the plating group [median (IQR): 7.0 (6.0–8.0) versus 6.0 (5.0–7.0), *p* = 0.010]. No significant differences were found in MSTS [28 (27–29) versus 27 (26.5–28.5), *p* = 0.23] and Karnofsky performance status scale scores [80 (70–80) versus 80 (70–80), *p* = 0.93] between groups. No proximal screw loosening was observed in the nailing group during the follow-up period, indicating that rigid fixation had been achieved. One patient in the nailing group had radial nerve palsy, while three patients in the plating group had radial nerve palsy and one had humeral head collapse and fragmentation without needing revision surgery. No complications, local recurrence, or need for revision surgery were reported in either group up until the last follow-up visit. Kaplan–Meier survival analysis for overall survival is shown in Fig. 2. No statistically significant differences were shown in the survival rate between the nailing group and the plating group (log-rank test *p* = 0.215).

**Table 2:** Comparison of outcomes between surgical procedures

Variable	Total	Nailing (*n* = 17)	Plating (*n* = 28)	*p*-Value
Surgical condition				
Blood loss, ml	300.0 (120.0–550.0)	100.0 (60.0–200.0)	500.0 (350.0–600.0)	** < 0.001**
Surgical time, min	111.0 ± 22.2	111.5 ± 17.5	110.7 ± 24.9	0.913
Length of hospital stay, days	10.8 ± 4.2	8.4 ± 2.6	12.3 ± 4.3	** < 0.001**
Complications	1 (2.2)	0 (0.0)	1 (3.6)	1.000^b^
Radial nerve injury	4 (8.9)	1 (5.9)	3 (10.7)	1.000^b^
Pain relief and function score				
VAS score				
Before surgery	8.0 (8.0–9.0)	8.0 (8.0–9.0)	8.0 (7.5–9.0)	0.881
One month after surgery	2.0 (2.0–2.0)	2.0 (0.0–2.0)	2.0 (2.0–2.5)	**0.002**
Reduction score (before–after)	6.0 (6.0–7.0)	7.0 (6.0–8.0)	6.0 (5.0–7.0)	**0.010**
Limitation of shoulder motion	15 (33.3)	5 (29.4)	10 (35.7)	0.664
MSTS score^a^	28.0 (27.0–29.0)	28.0 (27.0–29.0)	27 (26.5–28.5)	0.232
Karnofsky score	80.0 (70.0–80.0)	80.0 (70.0–80.0)	80.0 (70.0–80.0)	0.926
Survival status				
Follow-up time, months	18.0 (17.0–18.0)	18.0 (16.0–18.0)	16.5 (16.0–19.0)	0.488
Dead	21 (46.7)	10 (58.8)	11 (39.3)	0.203

*P*-Values < 0.05 are shown in bold

*MSTS* Musculoskeletal Tumor Society rating scale; *VAS* visual analog scale

a. MSTS score was checked at 3 months in patients still alive

b. Fisher’s exact test

## Discussion

This is the first study to compare the functional outcomes between nailing and plating in treating metastatic pathological fractures in the proximal humerus. Satisfactory outcomes were displayed in both surgical groups. Significantly more improvement of pain was reported in the nailing group, illustrating better pain relief by the use of humeral nails. Less blood loss and shorter hospital stay were also found in the nailing group compared with the plating group, showing that patients benefit from the less invasive procedure of the modern proximal humerus nail.

Intramedullary nailing was used less often for treating proximal humeral fractures decades ago because it did not provide rigid fixation, especially in patients with poor bone quality or osteoporosis. With the modern advancements in nail design, the nailing procedure has been used successfully in treating proximal humerus fracture [8–11] and pathological fractures of humeral shaft with good outcomes [12–’]. Intramedullary nailing with cement augmentation provides immediate stabilization of the fracture site and effective pain relief [13, 14]. Disadvantages are all associated with rotator cuff tendonitis, shoulder stiffness, and impingement [9]. In the present study, no rotator cuff impingement or shoulder stiffness was reported during follow-up, probably because we buried the nail end below the bony surface in order to prevent nail impingement, given that there is usually no need for nail removal in advanced-stage patients such as those in the present study. Younis et al. also reported that unreamed nailing tends to contribute to less blood loss and fewer complications without differences in the consolidation rate compared with reamed nailing [15].

Plate fixation has shown good outcomes in previous studies for stabilizing impending or complete pathological fractures in the humerus [5, 16–18]. Depending on the configuration, plating can be used in any humerus region. Still, the key to successful plate fixation is to allow for adequate proximal and distal cortical bone stock. Plate fixation displays similar functional status and complication rates compared with intramedullary nailing in treating diaphyseal pathological fracture, although it is associated with significantly higher blood loss [16]. Those results are similar to results of the present study, in which the plating groups had higher blood loss than the nailing group.

When the articular surface has been destroyed and the tumor is not confined, endoprosthesis has been regarded as the best choice. Endoprosthesis has the advantages of providing resistance to bending and rotation, good pain relief, local tumor control, and stability. However, the disadvantages are the need for 6–8 weeks of healing in a sling, rotator cuff repair, shoulder immobilization, or sometimes head subluxation. Kapur et al. reported that reverse shoulder arthroplasty provides good outcomes in treating proximal humeral metastatic diseases, including pain relief, restoration function, and no need to consider rotator cuff musculature [19]. Studies reveal that both nailing and plating have better functional recovery, pain relief, and no or few postoperative complications compared with conventional endoprothesis in treating pathological fractures of the proximal humerus [5, 6, 20–23] (Table 3). Taking the findings of the present study together, intramedullary nailing is more strongly recommended than locking plate because of greater pain relief, low blood loss, and shorter hospital stay, even while functional status and complication rates remain comparable.

**Table 3:** Summary of studies in the systematic review

Study	Mode of reconstruction	Sample size	Mean age (years)	Function	Pain control	Complications	Tumor recurrence	Reoperation
Piccioli et al. [21]	Endoprosthesis replacement	30	N/A	MTST score 73%	Intermediate (MTST pain: 4.75)	Infection: 2, nerve palsy: 1	3	0
Scotti et al. [22]		40	67 (52–75)	Enneking score 73.1%	N/A	Infection: 2	4	4
Tai et al. [23]		22	N/A	Restricted function: 8 (36%)	Persistent pain: 3 (13%)	Prosthesis migration: 1	0	0
Bickel et al. [20]		18	N/A	Satisfactory: 15 (83%)	Satisfactory	N/A	0	N/A
Siegel et al. [5]	Plating with cement	32	52.1 (38–79)	MTST score 94.6%; return to work without restriction: 22 (69%)	Intermittent mild pain on abduction > 90°: 8 (25%)	0	4	4
Choi et al. [6]	IM nailing with cement	32	59.8 (36–86)	MTST score 92%; Karnofsky score 75.6	Persistent pain: 1 (3.1%)	0	0	0

*MSTS* Musculoskeletal Tumor Society rating scale; *N/A* not available

The present study has several limitations. First, the sample size is small. Second, the retrospective study design has inherent limitations such as not allowing selection bias to be ruled out and limiting the generalization of results to other populations. Third, the follow-up period was short owing to the frequency of short survival in patients with pathological humerus fracture. A larger cohort study with long-term follow-up that compares postoperative outcomes between plating, nailing, and even endoprosthesis is warranted to provide more precise guidance for treating proximal humerus head and neck tumors, either confined or nonconfined.

## Conclusions

Both intramedullary nailing and plating are safe and effective surgical methods for treating metastatic lesions in the proximal humerus. Intramedullary nailing with cement augmentation is an option because it provides rigid fixation and allows early motion as a result of pain relief, which optimizes patients’ functional outcomes. Compared with plating, nailing is associated with less blood loss and shorter hospital stay.

## Data Availability

All data generated during this study are included in this published article.

## Abbreviations

MSTS: Musculoskeletal Tumor Society rating scale
VAS: Visual analog scale
